# Effects of Five-Year Treatment with Testosterone Undecanoate on Metabolic and Hormonal Parameters in Ageing Men with Metabolic Syndrome

**DOI:** 10.1155/2014/527470

**Published:** 2014-02-12

**Authors:** Davide Francomano, Andrea Lenzi, Antonio Aversa

**Affiliations:** Department of Experimental Medicine, Medical Pathophysiology, Food Science and Endocrinology Section, Sapienza University of Rome, Viale Regina Elena 324, 00161 Rome, Italy

## Abstract

Metabolic and hormonal modifications after long-term testosterone (T) treatment have never been investigated. 20 hypogonadal men (mean T = 241 ng/dL–8.3 nmol/L) with metabolic syndrome (MS, mean age 58) were treated with T-undecanoate injections every 12 weeks for 60 months. 20 matched subjects in whom T was unaccepted or contraindicated served as controls. Primary endpoints were variations from baseline of metabolic and hormonal parameters. In T-group, significant reductions in waist circumference (−9.6 ± 3.8 cm, *P* < 0.0001), body weight (−15 ± 2.8 Kg, *P* < 0.0001), and glycosylated hemoglobin (−1.6  ±  0.5%, *P* < 0.0001) occurred, along with improvements in insulin sensitivity (HOMA-I; −2.8  ±  0.6, *P* < 0.0001), lipid profile (total/HDL-cholesterol ratio −2.9 ± 1.5, *P* < 0.0001), systolic and diastolic blood pressure (−23 ± 10 and −16 ± 8 mm Hg, *P* < 0.0001, resp.), and neck and lumbar T-scores (+0.5 ± 0.15 gr/cm^2^, *P* < 0.0001; +0.7 ± 0.8, *P* < 0.0001, resp.). Also, serum vitamin D (+14.0 ± 1.3 ng/mL, *P* < 0.01), TSH (− 0.9 ± 0.3 mUI/mL, *P* < 0.01), GH (0.74 ± 0.2 ng/mL, *P* < 0.0001), and IGF1 (105 ± 11 ng/mL, *P* < 0.01) levels changed in T-group but not in controls. Normalization of T levels in men with MS improved obesity, glycemic control, blood pressure, lipid profile, and bone mineral density compared with controls. Amelioration in hormonal parameters, that is, vitamin D, growth hormone, and thyrotropin plasma levels, were reported.

## 1. Introduction

Obesity, and particularly visceral fat excess, is associated with insulin resistance, hyperglycemia, atherogenic dyslipidemia, and hypertension as well as prothrombotic and proinflammatory states and with vitamin D deficiency [1]. Several papers have suggested that a significant relationship between low levels of testosterone (T) and the metabolic syndrome (MS) exists [2]. Also, epidemiological studies have found that low T levels are a predictor of mortality in elderly men [3]. In addition, increasing evidence is accumulating regarding inverse associations between the severity of features of the MS and plasma T [4]. An inverse relationship between waist circumference (WC), a surrogate of visceral obesity, and T levels exists [5], thus leading to hyperinsulinism and reduced levels of sex hormone binding globulin (SHBG) and luteinizing hormone (LH), and all these factors along with increased leptin contribute to the suppression testicular steroidogenesis [6]. Also, in centrally obese individuals, there is an overactivity of the corticotropin-releasing-hormone (CRH)—corticotropin (ACTH)—cortisol axis as speculated by pioneer work of Björntorp and coauthors who demonstrated that this increased activity may result in a suppression of the production of T and growth hormone (GH) [7].

The European male ageing (EMAS) study is the first epidemiological study suggesting an upper limit of 11 nmol/L (FT 220 pmol/L) as the one correct for treating testosterone deficiency syndrome (TDS) [8]. Despite the fact that in this study the reported prevalence of hypogonadism was low (17%), Corona et al. reported an incidence as high as 29.3% in obese men [9]. This can be explained by the fact that EMAS investigated a relatively healthy sample of the general population, whereas Corona assessed T levels in outpatients presenting with erectile dysfunction (ED). In fact, T substitution in men with such values determines significant improvement in body composition, as reported in several studies [10, 11]. If this may be considered the threshold T level for the appearance of major symptoms like erectile dysfunction or decreased sexual desire, this may not be true for reverting body composition and mineral density changes induced by TDS. As previously demonstrated by other authors, the improvement in metabolic parameters may require achievement of higher and sustained therapeutic levels of testosterone over the time [12]. Moreover, evidence exists suggesting that T regulates adipogenesis and therefore increases lean body mass and reduces fat mass thus regulating body composition [13]. Long-term hormonal and anthropometric variations during T replacement therapy (TRT) in men with metabolic syndrome have not been investigated in controlled studies.

Aim of this study was to evaluate the effects of TRT on metabolic and hormonal parameters in hypogonadal men with MS.

## 2. Patients and Methods 

### 2.1. Inclusion, and Exclusion Criteria

Forty patients, aged from 45 to 65 years, were enrolled into this prospective study. Patients were included in the study if they were between 45 and 65 years of age, had MS and/or type 2 diabetes mellitus (T2DM) defined by the International Diabetes Federation [14] and total serum T level below 320 ng/dL (11 nmol/L) or calculated free-T levels below 255 pmol/L (74 pg/mL) on two early morning separate days (between 8:00 and 11:00 a.m.) at least 1 week apart, and had at least two symptoms of hypogonadism. Patients were not included in the study in case of the following: use of TRT or anabolic steroids or any other hormone replacement therapy in the previous 12 months; history of prostate or breast cancer or other tumours; drug or alcohol abuse; blood coagulation alterations; symptomatic obstructive sleep-apnoea syndrome; haematocrit level ≥52% at baseline; age-adjusted elevated prostate-specific antigen (PSA) level or abnormal digital rectal examination (DRE) of prostate suspicious for cancer or severe symptomatic benign prostatic hyperplasia; an International Prostate Symptom Scale (IPSS) >13 at baseline; use of 5-*α*-reductase inhibitors; presence of any uncontrolled endocrine disorder including diabetes (HbA1c ≥9); presence of New York Heart Association III or IV heart failure; hepatic insufficiency; severe neurological and psychiatric disease; and patients requiring or undergoing fertility treatment. We also excluded men who had diseases potentially affecting the skeleton, such as chronic renal disease or malabsorption, or were taking medications or drugs affecting bone turnover including any vitamin supplementation or nutraceutics or more than three alcoholic drinks a day. All concomitant oral hypoglycemic, anti-hypertensive, and lipid-lowering medications were permitted if started within the previous 12 months and continued throughout the study without dose adjustments. Subjects were asked to maintain their usual physical exercise and lifestyle for the duration of the study. Written informed consent was obtained before commencement of the study according to Protocol and Good Clinical Practice on the conducting and monitoring of clinical studies and approved by our University Ethical Committee.

### 2.2. Primary Outcome Measures

The primary outcomes were variation from baseline of the metabolic, bone, and hormonal parameters. At baseline, every three (within the first year) and six (in the following 4 years) months, the following evaluations were assessed: general physical examination and anthropometric parameters (i.e., body weight (BW), height, BMI, and waist circumference (WC)), systolic and diastolic blood pressure, heart rate, blood samples for biochemical and hormonal analyses, and digital rectal examination (DRE). Every twelve months, BMD was calculated by using a whole-body dual-energy X-ray absorptiometry (DEXA-HOLOGIC QDR-1000) according to the instructions of the manufacturer and standardized procedures, and the individual bone mineral density (BMD) variation has been measured with a T-score [15]. Calibration with the manufacturer's spine phantom and quality control analysis were performed daily. The long-term precision error *in vitro* was 0.54% (phantom); short-term precision error *in vivo* was 1.2% for the lumbar spine and 2% for the femoral neck [16]. BMD was expressed in grams per square centimeter (g/cm^2^) and result expressed as T-score.

Fasting blood samples were tested for glucose, triglycerides, high-density lipoprotein cholesterol (HDL-C), and low-density lipoprotein cholesterol (LDL-C) at the hospital's clinical laboratories. Hormonal assessment included serum total T (TT) and LH, as measured by chemiluminescent microparticle immunoassay (CMIA, Architect System) (Abbott Laboratories, Abbott Park, IL, USA), with detection limit of 0,28 M, calculated free T (according to http://www.issam.ch/), sex hormone binding globulin (SHBG), estradiol, prolactin, thyroid stimulating hormone (TSH), growth hormone (GH), somatomedin-C (IGF1), insulin, and PSA were analyzed by immunometric assay based on chemiluminescence using an automated clinical chemistry analyzer (Immulite 2000, Diagnostic Product Corp., Los Angeles, CA, USA). To overcome seasonal variability, 25-hydroxy vitamin D (25OHD; ng/mL) was measured by chemiluminescent immunoassay always during the same season and each subject served as an internal control (ARUP Laboratory, Salt Lake City, UT; coefficient of variation (CV) 8.6–10.0%). HbA1c was measured by high performance liquid chromatography (Bio-Rad Laboratories, Hercules, CA, USA). To assess insulin sensitivity, we calculated the HOMA-I using the formula [fasting insulin in mU/L × fasting glucose in mmol/L]/22.5.

### 2.3. Modality of Treatment

After screening any patient for the presence of hypogonadism, twenty of 72 patients met the inclusion/exclusion criteria and entered into the study. Patients received TU (TRT group) administered intramuscularly at a dose of 1000 mg every 6 weeks for the first two injections and then every 12 weeks, according to recommendations, for a period of 60 months. Twenty patients not fulfilling inclusion/exclusion criteria or refusing TRT for personal reasons and preferring lifestyle changes as the primary treatment were observed throughout the time and served as controls. Due to severe overweight, most patients adhered to comply with a standard hypocaloric diet and slight changes in lifestyle that is, low/moderate walking at least three times per week. Each patient was assigned to a personalized nutritional program, consisting in a hypocaloric diet with a protein of 0.8–1 g/Kg of lean body weight, along with a personalized movement program, with recommendation of at least 60 minutes/week of aerobic exercise of low/moderate intensity (40% of maximum heart rate). Physical activity should have been distributed in at least 3 days/week, and there must be no more than 2 consecutive days without activity [17]. The patients were monitored for compliance with a personal diary indicating “yes” or “no” regarding the lifestyle changes prescriptions.

### 2.4. Safety

Safety parameters included DRE, PSA total and free, hemoglobin, hematocrit, liver, and kidney functions were monitored every three (within the first year) and six (in the following 4 years) months, respectively, according to previously published procedures [18].

Patients with the following clinical laboratory parameters were withdrawn either at the baseline or during the course of study: if hematocrits levels were >52%; PSA level increased >1.0 ng/mL above the baseline PSA if baseline PSA was <2.0 ng/mL; PSA levels increase >50% of the baseline PSA if baseline PSA was >2.0 ng/mL.

### 2.5. Statistical Analyses

Data were analyzed using *t*-tests (for single between-group comparisons), analysis of covariance (for between-group comparisons at specific time points, using baseline score as a covariate), and a mixed linear regression model on repeated measures data (for between-group comparisons across all time points) to analyze data for an Intent-to-Treat Group (including all subjects enrolled and treated in this trial with values imputed for their Last Observation Carried Forward (LOCF) for any subjects who did not complete the trial) and a Completer's Group (including only data from subjects who completed the trial per protocol). Data were expressed as means ± standard deviation when normally distributed, and as median (quartiles) when nonparametric. A *P* value < 0.05 was taken as statistically significant. Statistical analysis was performed using the computer statistical package SPSS 11.0 (SPSS Inc., Chicago, IL, USA).

## 3. Results

### 3.1. Metabolic and Hormonal Parameters

Demographic characteristics of the patients at baseline are shown in Table 1.

All patients included were hypogonadal because of metabolic disturbances, that is, metabolic syndrome and/or diabetes, and none had primary/secondary hypogonadism with alteration of gonadotropins (data not shown). As expected, at the end of the study, the values of TT were higher in the TRT compared to the control group (+9.1 ± 1.7 nmol/L, *P* < 0.0001) while estradiol levels showed a trend to increase (Table 2).

**Figure 1:**
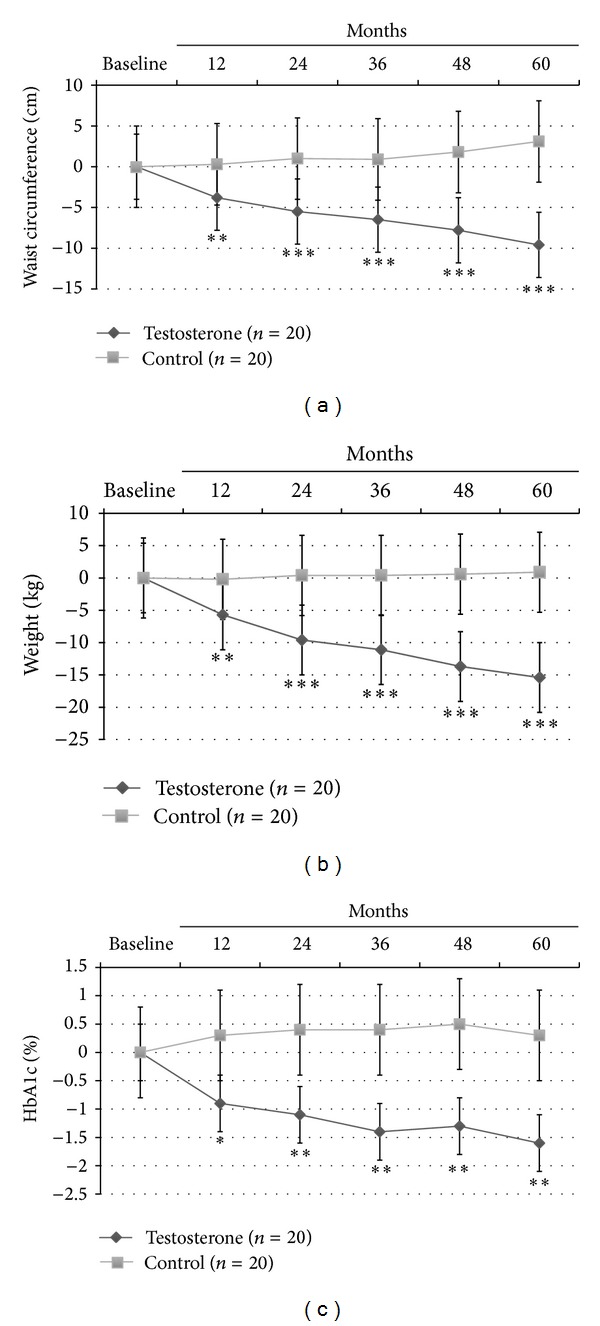
Effects of 5-year treatment with long-acting TU on (a) waist circumference (cm), (b) body weight (Kg), and (c) glucose homeostasis (HBA1c) in 40 hypogonadal men (T < 11 nmol/L) with metabolic syndrome (IDF). *P* variations were evaluated yearly in the testosterone treatment (TRT) versus controls (CTRL).

At LOCF, only TRT group showed a significant reduction of BMI (−2.9 ± 1.4, *P* < 0.0001); also, WC (−9.6 ± 3.8 cm, *P* < 0.0001; Figure 1(a)) and body weight (−15 ± 2.8 Kg, *P* < 0.0001; Figure 1(b)) significantly decreased in all men (100%) treated with TU compared with controls, who displayed a trend to increase both parameters over the time. This was mainly due to major compliance of TRT group towards diet and physical exercise compared with controls (90% versus 10% of overall patients, *P* < 0.0001, data not shown). There was a significant reduction of blood glucose as evaluated by mean HbA1c levels during the 60 months study follow-up period (−1.6 ± 0.5%, *P* < 0.001; Figure 1(c)) for the TRT group only.

**Figure 2:**
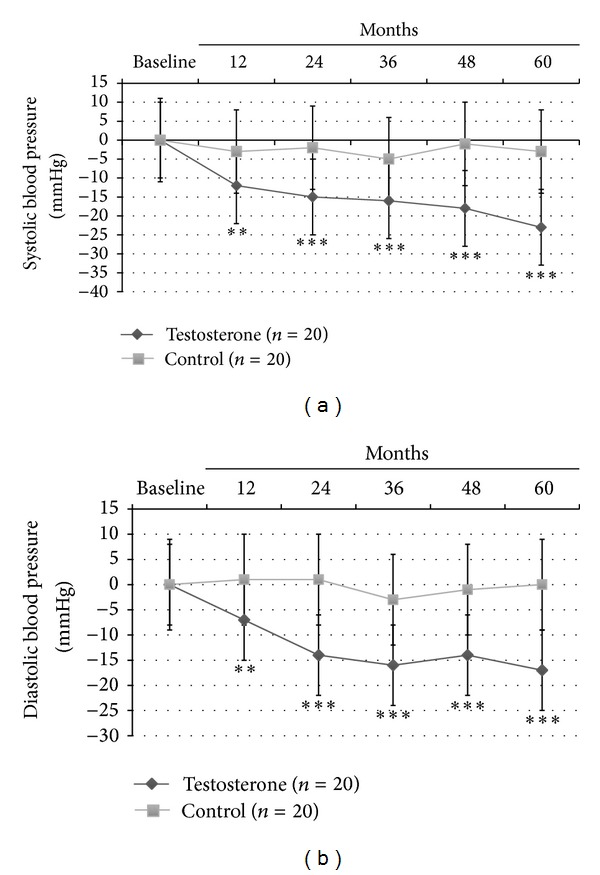
Effects of 5-year treatment with long-acting TU on (a) systolic blood pressure (mm Hg) and (b) diastolic blood pressure (mm Hg) in 40 hypogonadal men (T < 11 nmol/L) with metabolic syndrome (IDF). *P* variations were evaluated yearly in the testosterone treatment (TRT) versus controls (CTRL).

In this latter group, significant reduction in insulin sensitivity as evaluated by HOMA-i (−2.8 ± 0.6, *P* < 0.0001) and lipid profile (total/HDL-cholesterol: −2.9 ± 1.5, *P* < 0.0001; and Triglycerides: −41 ± 25, *P* < 0.0001) was found. Also only TRT group showed a significant reduction in both systolic (−23 ± 10 mm Hg, *P* < 0.0001; Figure 2(a)) and diastolic (−16 ± 8 mm Hg, *P* < 0.001; Figure 2(b)) blood pressure, heart rate (−15 ± 5 bpm, *P* < 0.001; Table 2) and a significant increment in neck and lumbar *T*-scores (+0.5 ± 0.15 gr/cm^3^, *P* < 0.0001; +0.7 ± 0.8 gr/cm^3^, *P* < 0.0001, resp.).

Interestingly, serum vitamin D (+14.0 ± 1.3 ng/mL, *P* < 0.01), TSH (−0.9 ± 0.3 mUI/mL, *P* < 0.01), GH (+0.74 ± 0.2, *P* < 0.0001), and IGF1 (+105 ± 11, *P* < 0.01) levels changed in TRT group only (Table 2).

### 3.2. Safety

A significant increase in hematocrit (+2.8 ± 0.9%, *P* < 0.001) and PSA levels (+0.37 ± 0.29 ng/mL, *P* < 0.01) within the normal reference range values was found in TRT group only without any clinical symptom or worsening in voiding function [19]. This increase occurred within the first 12 months of treatment and remained stable throughout the remaining period of study (Table 2).

## 4. Discussion

This is the first long-term controlled, nonsponsored study with T-undecanoate (TU) for a 60-month period in hypogonadal men with MS. Anthropometric, hormonal, and body composition parameters were investigated. Our results clearly demonstrate that TU is able to improve anthropometric measurements in a stepwise yearly manner, that is, WC and total BW; not surprisingly, a significant reduction in blood pressure and heart rate was reported compared to controls. Also, hormonal panel including vitamin D, TSH, GH, and IGF1 circulating levels all improved and these hormonal changes were not described elsewhere in such a population. No serious adverse event related to TU treatment was reported over the time.

Several recent studies have focused on normalizing T levels by using TU injections in obese hypogonadal men with TDS. Saad et al. investigated the effects of TU injection in 110 elderly men with obesity and MS and demonstrated that age, BMI, and C-reactive protein (CRP) levels, in addition to hypogonadism, can be used clinically to predict which men mostly benefit from T supplementation with regard to components of the MS [20]. Aversa et al. demonstrated that three-years TU in middle-aged men with TDS and MS determined a significant increase in both vertebral and femoral BMD that was correlated with the increments in serum T levels, probably independently from estradiol modifications and this was mainly related to CRP reduction [21]. In another study, Saad et al. demonstrated that TU treatment of 255 hypogonadal men determined a weight loss in approximately 95% of all patients, with marked changes in body composition, that is, an increase in lean body mass and a decrease in fat mass [22]. Yassin and Doros confirmed same results in a registry study of 261 hypogonadal men [23]. In all reported studies to date, T treatment consistently showed decreased fat and increased lean body masses. Similarly, Traish et al. reported significant changes in MS components during TRT at physiological levels [24]. Even if obtained in uncontrolled studies, these findings suggest that T may be a physiological modulator of body composition due to its role in promoting myogenesis and inhibiting adipogenesis and its role in carbohydrate, lipid, and protein metabolism. Data obtained in the present controlled study are confirmative of the evidence previously reported in uncontrolled studies that features of the MS present in elderly men must not be a limiting factor in prescribing TU in view of its advantages on metabolic, bone, and hormonal ameliorations as well as on overall improvements in estimated cardiovascular disease (CVD) risk.

T is a well-known regulator of many metabolic functions in liver, adipose tissue, muscles, coronary arteries, and the heart. The TC/HDL-C ratio is another important marker of CVD risk and its modification during treatment may indicate major changes in metabolic function that is, improvement in insulin resistance and decreased ischemic heart disease risk [25]. It is thought that it may represent a better marker than the apoB/apoA1 ratio for identifying insulin resistance and MS in some populations [26]. A recent study demonstrated that patients with peripheral artery disease treated with atorvastatin showed improvement in endothelial function and this was associated with decreased TC/HDL-C ratio, suggesting that this ratio may be related to endothelial damage [27]. The improvement of endothelial function may be the basis for the reduction of blood pressure and heart rate found in the present study. In fact, in previous report from our group we demonstrated that one-year TU is able to improve arterial stiffness and endothelial function in morbidly obese men (unpublished data), thus confirming that a sustained and advantageous effect of TRT on cardiovascular function is present in men with MS, thus leading to reduced CV risk throughout the time. The present data confirm, in a controlled study, that long-term TU reduces the risk of CVD in men with MS as previously described in observational studies [28].

Morbidly obese patients have been reported to often present with vitamin D insufficiency and secondary hyperparathyroidism. In obese women who undergo weight loss therapy, an abnormal vitamin D metabolism is still reported after 5-year follow-up [29]; similarly, bariatric surgery does not completely revert preexisting vitamin D deficient states and secondary hyperparathyroidism [30]. The reduction in WC and BW during weight loss program appear to be a common finding in the obese population following controlled weight loss programs; however, in our obese hypogonadal male patients (with MS), the finding of persistent and sustained yearly weight loss over the time was very surprising when compared with control group in whom no modification occurred despite the fact that slight lifestyle changes were recommended to both groups. Hagenfeldt et al. firstly described the improvement in vitamin D plasma levels after TRT in a small group of men with Klinefelter's syndrome through a possible, indirect action of increased estradiol circulating levels due to aromatization [31]. Other authors have speculated that, in normal conditions, Leydig cell may contribute to the 25-hydroxylation of vitamin D through the CYP2R1 enzyme that catalyzes the hydroxylation of cholecalciferol to 25-hydroxyvitamin D [32]. This enzyme is in turn regulated by insulin-like 3 (INSL3), which has also a role in osteoblast function, through an LH-T related mechanism. Testicular dysfunction determines reduced T levels, along with low INSL3 and 25-hydroxyvitamin D levels, and consequently may lead to an increased risk of osteopenia and osteoporosis. In our patients a mild osteopenia was present, and improvements in bone mineral density were reported despite no modification in estradiol levels. We speculate that the increase in vitamin D obtained by our patients may be partly due to T-induced overall trunk fat mass reduction, since in cross-sectional studies we had previously demonstrated a close relationship between trunk fat mass, vitamin D, osteocalcin, and testosterone levels in obese men [1]. Also, a direct effect of testosterone on renal expression of the l-alpha-hydroxylase gene might be possible, as androgen receptors have been demonstrated in kidney tissue [33].

On the other hand, other hormones or regulatory factors could mediate the effect on vitamin D indirectly. GH and IGF-I have been reported to influence vitamin D metabolism both in animals and in humans [34]. Previous studies demonstrated that increasing serum T concentrations to the mid-normal range with low-dose T administration for 26 weeks increases nocturnal, spontaneous, pulsatile GH secretion, and morning IGF-I concentrations in healthy older men, supporting the hypothesis that age-related reductions in T may contribute to the concurrent “somatopause” [35]. Accordingly, in the present study, the stimulatory effects obtained after TRT on GH secretion may be interpreted as an indirect effect due to the activation of lipolytic cascade of adipocytes leading to a better insulin sensitization, reduction of abdominal fat, and amelioration of pituitary function. Several reports in the literature consider obesity as a sort of “panhypopituitarism” condition determining a multiendocrine dysfunction. It is well established that caloric restriction applied for a relatively short term usually is able to increase GH release significantly in normal weight subjects [36]; however, this release results significantly reduced in obese subjects, who exhibit large diet-induced weight losses [37]. The recovery of the GH/IGF-I axis after weight loss suggests an acquired defect, rather than a preexisting pituitary disorder. Noteworthy, in our control group, we hypothesize that the persistent impairment of endocrine axes, that is, GH/IGF-I might have acted toward expansion and maintenance of fat mass and have contributed to perpetuation of the obese state.

Few studies have investigated the effects of controlled weight loss on thyroid hormone axis in male obese subjects. Cross-sectional studies have demonstrated that T3 and TSH correlate positively with adiposity [38]. In a recent study, moderate weight loss intervention resulted in a significant decrease in circulating T3 and only a marginal decrease in TSH and in fT4 [39]. Altogether, these observations indicate that even a moderate weight loss intervention may generate some perturbation in this axis. Our data obtained in TRT group clearly show that the stepwise decrease in fat mass, anthropometric and blood pressure parameters throughout the time may be considered an important factor also impacting on thyroid homeostasis. The fact that these changes were not observed in the control group is in keeping with the failure in achieving a correct weight (and abdominal fat) loss.

A limitation of the study represented by the low number of subjects investigated. We understand that it is difficult to rely on overall changes occurring in a small cohort of patients, but we are aware of the fact that this is a spontaneous, unsponsored study not designed to specifically investigate the effects of T on metabolic and hormonal pattern; thus patients were followed up for their specific comorbidities. Another limitation of this study was that a limited number of plasma hormones was investigated; thus PTH, gonadotropins, osteocalcin, and free fraction of thyroid hormones were not measured in all patients, in part because of financial constraints.

The marked weight loss observed in hypogonadal men with MS replaced with TU is an important finding of the present study and is in agreement with previous *in vitro* studies where T regulates lineage of mesenchymal pluripotent cells by promoting the myogenic lineage and inhibiting the adipogenic lineage [40]. T also inhibits triglyceride uptake and lipoprotein lipase activity resulting in rapid turnover of triglycerides in the subcutaneous abdominal adipose tissue and mobilizes lipids from the visceral fat depot [41]. Thus, T-induced changes on metabolism and body composition might have been determined by increased motivation, enhancement of mood, and promotion of more energy expenditure; this in turn might be responsible of the multiple endocrine modifications occurred on pituitary function. The changes in vitamin D levels and hormonal status (GH, IGF1, and TSH) are likely to be explained by the reduction of trunk fat mass content. By contrast, in control groups all these changes were not present despite the fact that lifestyle changes were applied.

In conclusion, this study demonstrates that TU in hypogonadal men with MS has favorable effect on body composition and metabolic parameters, after five-years replacement. The present study also provides first evidence that remarkable reduction of blood pressure and heart rate, as well as amelioration of vitamin D, GH/IGF1, and TSH plasma levels, are also attained. This may in turn yield to different overall CVD estimated risk and overall survival rates as well as to different pharmacological management of T2DM, hypertension, and dyslipidemia in men with MS and obesity.

## Figures and Tables

**Table 1:** Demographic characteristics of patients at baseline.

	Control (*n* = 20)	Treatment (*n* = 20)
Age (years)	57 ± 8	58 ± 10
BMI (kg/m^2^)	31 ± 6	31 ± 5
Only metabolic syndrome (*n*/%)	14 (70%)	14 (70%)
MetS + type 2 diabetes (*n*/%)	6 (30%)	6 (30%)
Smokers (*n*/%)	4 (20%)	4 (20%)
Treatments		
None (*n*/%)	8 (40%)	6 (30%)
Metformin (*n*/%)	8 (40%)	8 (40%)
Antihypertensives (*n*/%)	12 (60%)	8 (40%)
Statins (*n*/%)	4 (20%)	5 (25%)
Fibrates (*n*/%)	0 (0%)	2 (10%)
Other (*n*/%)	4 (20%)	5 (25%)

**Table 2:** Effects of five-year testosterone undecanoate treatment on anthropometric and hormonal parameters in 40 hypogonadal men with metabolic syndrome. *P* variations were evaluated yearly in the testosterone treatment (TRT) versus controls (CTRL).

	Baseline			12 months			24 months			36 months			48 months			60 months		
	CTRL	TRT	*P*	CTRL	TRT	*P*	CTRL	TRT	*P*	CTRL	TRT	*P*	CTRL	TRT	*P*	CTRL	TRT	*P*
Tot-Chol./ HDL-Chol.	4.9 ± 3.8	5 ± 3.5	ns	5.6 ± 2.7	2.7 ± 2.3	0.001	5.2 ± 2.8	2.3 ± 2.1	0.0001	5.1 ± 2.4	2.4 ± 2.2	0.0001	5.5 ± 2.0	2.17 ± 2.0	0.0001	4.8 ± 2.3	2.1 ± 1.5	0.0001
Trigl. (mg/gL)	187 ± 28	196 ± 31	ns	193 ± 21	167 ± 21	0.001	197 ± 24	172 ± 22	0.0001	181 ± 25	151 ± 21	0.0001	189 ± 23	147 ± 19	0.0001	182 ± 23	155 ± 19	0.0001
Heart rate (bpm)	89 ± 10	87 ± 10	ns	87 ± 9	82 ± 8	0.001	88 ± 9	78 ± 7	0.001	88 ± 7	76 ± 5	0.0001	87 ± 2	75 ± 5	0.001	88 ± 9	72 ± 5	0.001
BMI (Kg/m^2^)	31 ± 6	30.5 ± 5.5	ns	29.9 ± 6	28.2 ± 3.1	ns	30 ± 5.5	27.5 ± 3.3	ns	30 ± 4.4	27.3 ± 3.9	ns	29.2 ± 4.4	27.9 ± 4.2	ns	30 ± 4.4	27.6 ± 4.1	ns
HOMA-I	4.25 ± 0.3	4.2 ± 0.3	ns	4.05 ± 0.3	2.1 ± 0.3	0.0001	3.65 ± 0.5	2.13 ± 0.4	0.0001	3.35 ± 0.4	1.7 ± 0.6	0.0001	3.35 ± 0.7	1.6 ± 0.6	0.0001	3.15 ± 0.6	1.4 ± 0.6	0.0001
VIT D (ng/mL)	18.4 ± 9.9	15.1 ± 8.6	ns	17.8 ± 9.7	25.3 ± 6.4	0.01	20.7 ± 8.1	26 ± 5.2	0.01	17 ± 7.8	28.5 ± 6.5	0.01	18 ± 6.7	30.3 ± 7.4	0.01	16.8 ± 8.1	29.1 ± 7.3	0.01
Total T (nmol/L)	9 ± 1.7	8.3 ± 2.4	ns	9.35 ± 1.4	15.9 ± 1.4	0.0001	8.6 ± 1.2	16.8 ± 1.7	0.0001	7.9 ± 0.8	17.6 ± 1.5	0.0001	8.1 ± 1.6	16.9 ± 1.7	0.0001	8.7 ± 1.4	17.4 ± 1.7	0.0001
SHBG (nmol/L)	34 ± 10	30 ± 13	ns	35 ± 13	31 ± 11	ns	31 ± 12	29 ± 9	ns	36 ± 11	28 ± 10	ns	34 ± 14	28 ± 9	ns	35 ± 12	28 ± 8	ns
Estradiol (pg/mL)	30 ± 9	26.5 ± 11	ns	29 ± 6	32 ± 11.5	ns	26 ± 7	31.5 ± 10	ns	28 ± 8	29.5 ± 9	ns	29 ± 7	32.5 ± 10	ns	34 ± 7	32.5 ± 10	ns
TSH (mUI/mL)	1.7 ± 0.3	2 ± 0.8	ns	1.9 ± 0.4	1.1 ± 0.5	0.01	2 ± 0.3	1.1 ± 0.4	0.01	2.2 ± 0.2	1.3 ± 0.3	0.01	1.9 ± 0.2	1.0 ± 0.4	0.01	2.5 ± 0.5	1.1 ± 0.3	0.01
GH (ng/mL)	0.20 ± 0.1	0.31 ± 0.3	ns	0.25 ± 0.1	0.95 ± 0.2	0.0001	0.25 ± 0.1	0.98 ± 0.1	0.0001	0.32 ± 0.1	1.0 ± 0.1	0.0001	0.4 ± 0.2	1.12 ± 0.2	0.0001	0.22 ± 0.1	1.05 ± 0.2	0.0001
IGF1 (ng/mL)	180 ± 43	157 ± 31	ns	188 ± 23	215 ± 22	0.01	189 ± 35	252 ± 23	0.01	195 ± 35	241 ± 27	0.01	140 ± 6	251 ± 18	0.01	177.5 ± 7	262 ± 20	0.01
Tot. PSA (ng/mL)	0.98 ± 0.25	1.05 ± 0.2	ns	1.05 ± 0.27	1.36 ± 0.31	0.01	1.03 ± 0.2	1.35 ± 0.2	0.01	1.0 ± 0.2	1.34 ± 0.3	0.01	1.02 ± 0.2	1.37 ± 0.2	0.01	1.04 ± 0.2	1.42 ± 0.3	0.01
HCT (%)	42.5 ± 0.3	43.8 ± 0.2	ns	41.9 ± 0.2	46.1 ± 0.8	0.001	41.8 ± 0.3	46.1 ± 0.7	0.001	43 ± 0.3	46.4 ± 0.6	0.001	41.1 ± 0.7	46.5 ± 0.6	0.001	43.5 ± 0.3	46.6 ± 0.9	0.001
Lumbar T-score (SD)	−1.6 ± 0.8	−1.6 ± 0.9	ns	−1.6 ± 0.7	−1.4 ± 0.8	0.05	−1.7 ± 0.6	−1.2 ± 0.8	0.005	−1.7 ± 0.8	−1.0 ± 0.8	0.0001	−1.9 ± 0.6	−1.1 ± 0.9	0.0001	−1.8 ± 0.7	−0.9 ± 0.8	0.0001
Neck T-score (SD)	−0.9 ± 0.8	−0.9 ± 0.8	ns	−0.9 ± 0.7	−0.7 ± 0.7	0.05	−0.9 ± 0.7	−0.6 ± 0.7	0.005	−1 ± 0.8	−0.5 ± 0.7	0.0001	−1.1 ± 07	−0.4 ± 0.7	0.0001	−1.3 ± 0.7	−0.4 ± 0.6	0.0001
